# Stigma of Palliative Care among Patients with Advanced Cancer and Their Caregivers on Early Palliative Care

**DOI:** 10.3390/cancers15143656

**Published:** 2023-07-18

**Authors:** Elena Bandieri, Eleonora Borelli, Fabio Gilioli, Sarah Bigi, Claudia Mucciarini, Umberto Ferrari, Sonia Eliardo, Lidia Pinto, Carlo Adolfo Porro, Fabio Efficace, Mario Luppi, Leonardo Potenza

**Affiliations:** 1 Oncology and Palliative Care Units, Civil Hospital Carpi, Azienda Unità Sanitaria Locale, 41012 Carpi, Italy; 2 Department of Medical and Surgical Sciences, University of Modena and Reggio Emilia, 41124 Modena, Italy; 3 Department of Internal Medicine and Rehabilitation, Azienda Unità Sanitaria Locale, 41121 Modena, Italy; 4 Department of Linguistic Sciences and Foreign Literatures, Catholic University of the Sacred Heart, 20123 Milan, Italy; 5 Department of Biomedical, Metabolic and Neural Sciences, University of Modena and Reggio Emilia, 41125 Modena, Italy; 6 Center for Neuroscience and Neurotechnology, University of Modena and Reggio Emilia, 41125 Modena, Italy; 7 Health Outcomes Research Unit, Italian Group for Adult Hematologic Diseases (GIMEMA), 00161 Rome, Italy; 8 Hematology Unit, Azienda Ospedaliera Universitaria di Modena, 41124 Modena, Italy

**Keywords:** early palliative care, stigma, advanced cancer, patients, caregivers

## Abstract

**Simple Summary:**

Early palliative care represents a successful model of care for advanced cancer patients and their caregivers. Yet, early palliative care provision remains confined to the last weeks of life. Among the possible reasons, the stigma associated with the name “palliative care” seems to have a prominent role. The present study aimed to investigate the perception of palliative care that a sample of 78 patients and 110 caregivers had before their referral to the early palliative care service. The results suggest on which levels it is necessary to intervene to overcome the stigma. From a policy perspective, it is clear that broad education is needed to ensure a more widespread understanding of the essence of anticipated palliative care.

**Abstract:**

The early referral to palliative care (PC) represents a successful value-based model with proven benefits regarding the quality of life and clinical outcomes for advanced cancer patients and their caregivers. Yet, its provision remains typically confined to the last weeks of life as per the historical, late PC model. The stigma according to which PC represents end-of-life care has been identified as the root of the problem. To explore the presence and effects of the stigma in a clinical context, we surveyed 78 patients and 110 caregivers (mean age: 71.7 and 60.7, respectively) on early PC to study what their perception of PC was before their direct experience. The responses were analyzed through a qualitative descriptive approach. The participants explicitly mentioned a lack of knowledge about PC (53% of the sample), which they identified also among physicians and the population (13%); an identification of PC with the late PC model (53%); and a detrimental reaction to the proposal of an early PC referral (83%). However, the participants explicitly mentioned that a direct experience of early PC allowed for an acquired awareness of early PC meaning and benefits (52%), as well as a comprehension of its differences with late PC (34%); the regret for the delayed referral (8%); the perception of the word “palliative” as a barrier (21%); and the belief that early PC should be part of the cancer routine practice (25%). A comprehensive multi-level intervention is necessary for a widespread understanding of the essence of anticipated PC.

## 1. Introduction

The early integration of palliative care (PC) into standard oncology care represents an innovative value-based model with proven short- and long-term benefits on quality of life and clinical outcome, including life expectancy, for advanced cancer patients with a prognosis of 6–24 months and their caregivers [1,2,3,4,5,6,7,8,9,10].

PC has evolved since the 1960s, from an initial effort focused on end-of-life cancer and hospice care [11] to a solicitation that “the principles of PC should be applied as early as possible in the course of any chronic illness, at the end fatal” [12]. The main oncology guidelines recommend early PC as a standard of care in advanced cancer, to be activated within 8 weeks of diagnosis, simultaneously with active oncological treatments [13,14,15,16]. However, its provision still remains confined to the last weeks of life, as per the historical, late model [11,13], with a referral of 19 days on average before death [14]. Noteworthy, efforts to anticipate the offer of PC to patients with advanced cancer and hematologic malignancies have been recently described in real-life settings [17,18].

Therefore, it is critical to identify which barriers prevent the timely adoption of PC.

One possible obstacle may be associated with the lack of PC physicians or PC units in hospitals [15]. Yet, even in oncology and hematology centers with established PC services, they are frequently underused [15,16]. As emerged from focus groups with patients, caregivers, and healthcare providers, the main barrier in preventing the timely adoption of PC seems to be associated with the negative connotation of the name PC itself, which acts as a deterrent in early referral from providers and early demand from patients [19,20]. The origin of this negative connotation is likely due to the stigma according to which PC represents the only care when any other intervention is no longer feasible [11,16,19,20,21,22,23,24]. Possibly, this misunderstanding arising in the original conceptualization of PC as end-of-life care is sustained by the oncologists’ perseveration in late referral [20,21,24,25,26,27,28]. Moreover, Western medicine has always been mainly focused on curing the disease and increasing life expectancy rather than improving quality of life and reducing suffering [23], leading to the wrong belief that a treatment is either curative or palliative and contributing to the stigma of PC as a care for those who are dying or “giving up”.

Because of this stigma, many physicians avoid early PC referral, worrying that patients and caregivers may be discouraged by a care proposal that, bringing to mind the thought of death, would take away their hope, induce negative emotions, and allow pessimism about the future and a feeling of abandonment by the medical team [19,29].

However, the literature on the perception of PC from public opinion, albeit scarce, provides a different picture from that feared by physicians. The public seems to have little to no idea of what PC is and does not attribute any particular value to it [30,31,32,33,34,35].

The fear of doctors regarding proposing PC seems, therefore, to be mostly unfounded and is likely influenced by the so-called “false consensus bias”, i.e., the tendency to project one’s thoughts onto others [36]. Accordingly, it has been shown that the knowledge that healthcare providers have of PC is often limited, distorted, and influenced by the stigma that has always accompanied it [26,37].

This hypothesis finds confirmation in the studies on patients’ and caregivers’ opinions on PC. In these studies, more than a lack of information, real disinformation emerged, and the influence of stigma was evident. PC was often linked to death, despair, addiction, and the end of life [28]. The patients also associated PC with a sense of failure and feelings of guilt and shame [38], as well as devaluation and a fear of being abandoned by the medical team [39]. In a grounded theory study by Zimmermann et al. [28], patients and caregivers from a cluster randomized trial on early PC associated PC with death, hopelessness, dependency, and end-of-life care. The participants reported feelings of fear and avoidance toward PC, often originating from interactions with healthcare professionals or from the media. However, the perception of PC in the intervention group dramatically changed, switching to that of ongoing care improving the quality of life [24]. Yet, they still felt that the name PC carried a stigma. The present study investigated the perception of PC among a sample of 188 advanced cancer patients and caregivers on early PC with the aim of understanding the pre-existing perceptions of this type of care before their exposure to it in a real-life setting.

The results showed the existence of a stigma associated with the name “palliative” among advanced cancer patients and caregivers, its deleterious effect in shaping the perception of PC, and the following reaction of fear and avoidance. The results also revealed how direct knowledge of the early PC service can restore the perception of the name and provide insights into the areas where interventions are needed to overcome the stigma.

## 2. Materials and Methods

### 2.1. Participants

We previously investigated several aspects of patients’ and caregivers’ experiences with early PC by administering a questionnaire to 133 advanced cancer patients and 118 caregivers of alive or deceased advanced cancer patients [40,41,42,43]. The participants were subsequently approached in person or by telephone by their palliativist or nurse and asked about their availability to answer one further open-ended question.

The patient and caregiver eligibility required a willingness to complete the task and age ≥ 18 years. All the participants provided written informed consent before the data collection. The study was performed in accordance with the ethical standards of the 2013 Declaration of 141 Helsinki and was approved by the Ethics Committee of Modena (N. 0026448/20).

### 2.2. Study Setting

The study was conducted in the outpatient Oncology and Palliative Care Unit, Civil Hospital Carpi, Azienda Unità Sanitaria Locale, Modena (Italy). The unit was established in 2006 and integrates primary oncology specialists with a palliative/supportive care team composed of one physician assistant, one fellow, and one nurse who specializes in PC to provide comprehensive symptom management and psychosocial and spiritual support to patients with cancer and their families, from the time of diagnosis to advanced/metastatic disease, according to established guidelines [2,9,44,45]. Patients with an advanced/metastatic cancer diagnosis (with distant metastases, late-stage disease, and/or a prognosis of 6–24 months) with a high symptom burden are electively referred by the oncologists to receive an early PC intervention. The early PC team follows, on average, 20–30 patients/week and each patient on a regular basis 1–2 times/week. The outpatients’ early PC interventions are integrated with both specialized nurse home care services and hospices [2,9,46].

### 2.3. Stimuli and Procedure

Once the patient and caregiver agreed to answer the further open-ended question, they were provided, in person or by e-mail, with the informed consent to be signed. They were then asked, “Before your access to this unit, what did the expression ‘palliative care’ mean to you?”. The open-ended question was posed in person or by telephone, based on the participant’s preference, by their palliativist or nurse, who transcribed the response as the participant answered orally. Once the question was answered, the palliativist or nurse asked the participant to confirm that the transcription corresponded to the intended answer. The interaction lasted a couple of minutes.

### 2.4. Data Analysis

Descriptive statistics were calculated to characterize the sample.

A qualitative descriptive approach, as detailed by Sandelowski [47], was adopted to analyze the responses in order to focus on actual words and manifest meanings instead of identifying possible latent significances behind statements and to limit the risk of experimenters’ interpretations or biases.

To identify the overall categories of responses to the open-ended question, two authors (EBa and EBo) independently applied a first-level coding and clustered the resulting codes through a second-level coding [48]. Subsequently, the two authors discussed their independent results to adjust them. The second-level code clustering allowed us to identify the main themes in the participants’ responses.

The identified main themes were qualitatively described and integrated with illustrative quotations. Percentage frequency analysis was used to quantitatively describe their occurrence. Since the object of the analysis was an open-ended question, to which the participants had to respond freely without the support of bullet points, the percentage frequency should be interpreted not as the percentage of participants who had a specific perception of PC but as the percentage of participants who verbalized it. 

## 3. Results

Compared to the original sample of 133 patients and 118 caregivers, 188 (74.9%) responses were collected, of which 78 were from patients and 110 were from caregivers. The remaining 55 patients were deceased at the time of the data collection, and 8 caregivers were not reachable by phone.

The descriptive statistics of the sample are reported in Table 1.

Twenty-four first-level code categories were identified and grouped into the following nine second-level code categories: (i) lack of knowledge about PC; (ii) identification of PC with late PC; (iii) early reaction to PC; (iv) detection of a lack of knowledge about PC also among physicians and the population; (v) acquired awareness of early PC meaning and benefits; (vi) awareness of the differences between early PC and late PC; (vii) regret for the delayed referral; (viii) name as a barrier; (ix) need to include early PC in the oncology clinical routine (Table 2).

Regarding the first category (lack of knowledge about PC), 53% of the participants reported not knowing the meaning of PC when it has been proposed by the oncologist, while 47% of them did not know it at all, with a few showing an open attitude toward the physician’s proposal. The remaining 53% reported not knowing the name yet to associate it with that of late PC or to a general negative valence.

The second category (identification of PC with late PC) was identified in 44% of the patients’ and 59% of the caregivers’ responses, who reported that the name matched with the idea of the end of life, death, and losing hope.

The third category (early reaction to PC) was identified in 38% of the patients’ and 44% of the caregivers’ responses, who reported that the name was perceived as negative, bad, or evoking feelings of fear and terror. The negative feelings associated with the name led to reactions like refusal, avoidance, and stalling.

The fourth category (detection of a lack of knowledge about PC also among physicians and the population) was identified in 5% of the patients’ and 18% of the caregivers’ responses, who hypothesized a diffused lack of knowledge regarding the existence and exact meaning of PC in the population and among oncologists. Some participants reported that they had previous experiences with family or friends with a terminal illness who had come into PC. In these situations, PC had generally been presented by health care professionals as an option of last resort.

The fifth category (acquired awareness of early PC meaning and benefits) was identified in 25% of the patients’ and 26% of the caregivers’ responses, who reported how they realized their idea of PC was wrong. The participants mentioned the benefits they gained from early PC, like pain control, improved quality of life, death acceptance, and possibly increased life expectancy. The patients and caregivers described early PC as hope and were grateful for it. One patient reported that early PC was the only reason he did not choose euthanasia. One element that was mentioned was the essential role early PC also had for caregivers.

The sixth category (awareness of the differences between early PC and late PC) was identified in 6% of the patients’ and 24% of the caregivers’ responses, who described early PC as life care instead of end-of-life care. Early PC was defined as being different from late PC, more than late PC, the opposite of late PC, and having nothing to do with late PC.

The seventh category (regret for the delayed referral) was identified in 3% of the patients’ and 5% of the caregivers’ responses, who reported a feeling of regret, were sorry, or were angry for not being referred to the PC service earlier.

The eighth category (name as a barrier) was identified in 10% of the patients’ and 11% of the caregivers’ responses, who pointed out how the stigma associated with the name is too strong to be overcome and how PC would be more widely diffused with a less stigmatized name. ‘Supportive care’ has been proposed as an alternative, followed by ‘pain care’.

The ninth category (need to include early PC in the clinical routine) was identified in 10% of the patients’ and 14% of the caregivers’ responses, who claimed that early PC should be an option for all cancer patients. Some stated that it should be mandatorily provided as a standard of care and that its integration into the oncology care routine could contribute to bypassing the stigma.

## 4. Discussion

In the present study, we investigated the perception of PC at the time of referral in a sample of 188 advanced cancer patients and caregivers.

The results highlighted the strong stigma associated with the word “palliative” and its possible deleterious effects on the acceptance of PC. They also revealed how direct knowledge of early PC can counteract such negative effects and restore its perception and provide insights into the areas where interventions are needed to overcome the stigma.

While some participants expressed uncertainty about the meaning of PC, saying they were not sure what the term exactly meant, and others saying they had no idea what it stood for, most of them described PC as carrying a negative meaning associated with death and the end of life, which resulted in fear and avoidance.

Interestingly, the participants often reported that they did not know about PC and that they identified it with end-of-life care. This apparent contradiction may be explained by assuming a diffuse scarce knowledge of PC in public opinion, as confirmed by the literature [30,31,32,33,34,35], and a concomitant attempt to fill this gap by borrowing the meaning of the word “palliative” from its everyday use (e.g., palliative solution, palliative option). It is interesting to note that although the word “palliative” has been coined in the clinical context to define end-of-life care based on the modern hospice movement [11], it is possible that people are more familiar with the adjective “palliative” in a non-clinical context more than in association with the word “care”.

The finding about the participants not knowing the exact meaning of PC yet attributing to it a negative valence is in accordance with a study by Shen and Wellman [23] specifically designed to detect the influence of the PC stigma among common people. Based on this study, the value attributed to PC and the influence of its stigma are modulated by the contextualization of the model itself. In other words, it is possible that a neutral or even positive perception of a model of care aimed at reducing pain at the end of life may gain a negative valence once placed in a clinical context with a range of alternative therapeutic options.

The uncertainty associated with a type of care that the participants did not know but perceived negatively led to fear and avoidance. Fear and avoidance have been an important and recurrent theme in our sample. This emotional and behavioral reaction could be overcome by the oncologist when proposing the referral to the PC service. However, as some participants explicitly said, even doctors were reluctant to propose a PC referral or they were not able to explain the benefits of an early referral. In fact, it was inadequate medical information that emphasized the stigma and the subsequent rejection of the early provision of PC. 

Interestingly, in agreement with the results of a recent study by Zimmermann et al. [24], our participants reported that their initial perception was restored by their direct experience of the service as well as comprehensive communication about the meaning of early PC from PC practitioners. After referral to the early PC service, the fear was generally replaced by positive feelings and attitudes toward it, especially with the enrollment exceeding 8–10 months, motivating patients to identify the early PC as an essential and unmissable appointment. The early PC benefits listed by our participants were those extensively reported in the literature, i.e., the resolution of physical and psychological pain [17,49], an improved quality of life leading to a feeling of gratitude thanks to the trustful and honest relationship with the health care team [41], lower incidence of negative emotions [40], and increased survival [44,50]. Surprisingly, early PC represented a sort of antecedent of hope for the patients and caregivers [42].

Notwithstanding the restored perception of PC after direct experience with its early provision, many participants felt that the word “palliative” still conveyed discomfort and that it was not the correct term to describe the care they were receiving. Based on their responses, the semantic and emotional dissonance of the word ‘‘palliative” was problematic, as also found by Cherny et al. [16]. A recurrent theme was that PC should be explicitly renamed. Several participants expressed a preference to either relabel the care they were receiving as “supportive” or to refer to their treating palliative physician as their “pain specialist”. A great variability of conflicting emotions in coming to terms with the idea of death made the transition from active care to PC, considered by all to be a surrender, very difficult. For this reason, the name PC for patients in the active phase of the disease was much opposed. The rebranding and renaming of PC were proposed by the participants to address the inconsistency between the early PC they had received and the persistent association of the name PC with end-of-life care.

The existence of the PC stigma has led some hospital centers to rename their PC programs to “supportive care programs”, a term that is perceived as less negative by users and that limits the discomfort of healthcare providers in proposing its early referral [19,51]. However, based on the work of some scholars, the two expressions are not interchangeable, and, in some cases, such as that of terminal patients in active treatment, the use of the expression “supportive care” is improper and misleading. Furthermore, replacing the name “palliative” with the name “supportive” may lead the doctor to avoid addressing the issue of the end of life [19].

In a 2001 editorial in the American Journal of Hospice and Palliative Medicine, Chamberlain [52] wrote, referring to the stigma associated with the term “hospice”, that the likely root problem, with an unpopular model of care, may be the name itself and that renaming it may solve the problem. The actual problem of the stigma associated with the name PC seems to be an ironic parody of the problem associated with its predecessor: the term “hospice”. Whichever term we choose will acquire the same negative value as the previous one if we do not intervene on different levels [27]. To progress in the de-stigmatization of PC we must educate the public, patients, caregivers, and institutions about what PC programs do. The public can be re-educated regarding the meaning that PC has acquired in recent years, eventually through media and social media [11]. It is crucial for institutions to offer PC as “part of the treatment package”, allowing patients and caregivers to bypass the stigma and any reflections on it. It is also of paramount importance to educate healthcare professionals about a broader understanding of the possibilities offered by PC and, in particular, by their early activation simultaneously with active oncological treatments [11,13].

A possible limitation of the study is found in a lack of the use of text analysis software, which may limit the ability to efficiently handle extensive datasets. However, manual analysis remains a valuable method for qualitative data analysis when dealing with short responses or when facing institutional policy restrictions in installing software, as in the present case. Employing rigorous coding protocols and maintaining transparency in the analysis process can still yield valid and reliable results.

A potential future direction for this study could be represented by a specifically detailed survey that would correlate responses with demographic and clinical/caregiving data. This approach would provide valuable insights into how different, individual factors may influence the perceptions of PC care and would contribute to more personalized care. This study contributes to the important perspective of patients with advanced cancers and their caregivers regarding their perception of PC before a direct experience with its early referral. The results showed that patients and caregivers have scarce knowledge of PC, yet the associated stigma negatively affects their perception, leading to a reaction of fear and avoidance. The patients and caregivers hypothesized that the same perception is shared by the public and physicians. A direct experience of early PC completely restored the idea of PC, yet rebranding the name is considered necessary to facilitate PC acceptance by the public and future patients.

## 5. Conclusions

Our study confirms that among advanced cancer patients and caregivers, the persistence of the negative and distressing stigma of PC is associated with death and end-of-life care. Despite an international shift in this definition more than a decade ago linked to the introduction of early PC, the stigma remains strong and unchanged. The results of our study have important implications for possible solutions to the problem. They confirm that, to contrast the stigma associated with the word “palliative” and restore the perception of PC, a multi-level intervention should be planned. At the “society” level, an easy-to-understand message regarding what early PC is and what benefits it brings should be conveyed through awareness-raising campaigns through various communication channels to reach a wider audience. A preliminary consideration of the pros and cons of a name change is mandatory. At the “healthcare providers” level, dedicated training programs should be incorporated into university curricula, and ongoing educational opportunities should be promoted. At the “institutional” level, the implementation of early PC units and their integration into standard treatment practices should be planned. Notwithstanding that a comprehensive intervention on different levels may counteract the deleterious consequences of the stigma, a direct experience of the service has also been found to be beneficial. Thus, personal stories and testimonials should be encouraged as powerful tools for spreading awareness.

## Figures and Tables

**Table 1:** Demographic and clinical/caregiving characteristics of the sample (n = 188).

			Patients (n = 78)	Caregivers (n = 110)
Age at enrollment	Years	Mean (sd)	71.7 (9.7)	60.7 (13.7)
		Range	43–88	23–85
Sex	Female	n (%)	37 (47.4)	73 (66.4)
	Male	n (%)	41 (52.6)	37 (33.6)
Education	Primary school	n (%)	26 (33.3)	9 (8.2)
	Secondary school	n (%)	22 (28.2)	25 (22.7)
	College	n (%)	28 (35.9)	47 (42.7)
	Graduation’s degree	n (%)	0 (0)	4 (3.6)
	Bachelor’s degree	n (%)	2 (2.6)	25 (22.7)
Ethnicity	Caucasian	n (%)	78 (100)	107 (97.3)
	African	n (%)	0 (0)	1 (0.9)
	Arabian	n (%)	0 (0)	2 (1.8)
Religion	Catholic	n (%)	65 (83.3)	81 (73.6)
	Muslim	n (%)	0 (0)	2 (1.8)
	Evangelic	n (%)	0 (0)	1 (0.9)
	Orthodox	n (%)	3 (3.9)	3 (2.7)
	Jehovah’s Witness	n (%)	1 (1.3)	1 (0.9)
	Animist	n (%)	9 (11.5)	2 (1.8)
	Atheist/Agnostic	n (%)	0 (0)	20 (18.2)
Cancer diagnosis	Breast	n (%)	11 (14.3)	-
	Colon	n (%)	7 (9.1)	-
	Gastric	n (%)	12 (15.6)	-
	Genitourinary (kidney, testis, prostate, ovary)	n (%)	20 (26)	-
	Head, neck, larynx	n (%)	2 (2.6)	-
	Lung	n (%)	14 (18.2)	-
	Pancreas	n (%)	5 (6.5)	-
	Rectum, sigma	n (%)	4 (5.2)	-
	Sarcoma	n (%)	1 (1.3)	-
	Blood	n (%)	2 (2.6)	-
Months on early PC at the time of study recruitment	Months	Mean (sd)	41.1 (10.4)	57.9 (17.9)
		Range	25–65	8–137
KPS score at first early PC consult	0–100	Median (IQR)	60 (60–70)	-
NRS pain score at first early PC consult	0–10	Median (IQR)	7 (6–8)	-
Active CT/RT at first early PC consult		n (%)	70 (89.7)	-
Status of the patient (alive/deceased) at the moment of the caregiver enrollment	Alive	n (%)	-	82 (74.6)
	Deceased	n (%)	-	27 (24.5)
	Missing data	n (%)	-	1 (0.9)
In the case of a deceased patient, months passed since the death	Months	Mean (sd)		47 (7.6)
		Range		38–70
Relationship to patient	Mother/Father	n (%)		1 (0.9)
	Spouse/Partner	n (%)		54 (49.1)
	Daughter/Son	n (%)		48 (43.6)
	Sister/Brother	n (%)		4 (3.6)
	Other family members	n (%)		3 (2.7)

Abbreviations: -, no data; CT, chemotherapy; PC, palliative care; IQR, interquartile range; KPS, Karnofsky Performance Status; NRS, Numerical Rating Scale; RT, radiotherapy.

**Table 2:** First- and second-level code categories and illustrative quotations from the responses to the open-ended question.

First-Level Categories	Second-Level Categories	Quotations
1. No idea of what it was	1. Lack of knowledge about PC	“I didn’t know it.” (002-C-069) “We did not know early palliative care before the oncologist proposed it to us. But having explained to us that it was intended for pain management, we immediately accepted it.” (002-C-049) “I don’t know, I was afraid of it because a friend of mine went to a place to receive this treatment and then died in a short time. I was afraid of ending up like that.” (002-P-110) “I was afraid of it because it seemed to me the care for when there is nothing more to do.” (002-P-136)
2. Vague/uncertain idea of what it was		
3. Identification with end-of-life care	2. Identification of PC with late PC	“(…) we thought it meant that there was nothing more to do.” (002-C-003) “To me, it was something negative, it was like saying there was no more hope, that you were already dead.” (002-P-109)
4. Synonymous of end of life/death		
5. Something/a word to be afraid of	3. Early reaction to PC	“To me, it sounded like something negative.” (002-P-109) “It was a bad word.” (002-P-087) “It was scaring.” (002-C-111) “It frightened me, they even terrified me (…).” (002-P-102) “I thought it was end-of-life care, (…) thus we refused it.” (002-C-017) “(…) I was afraid of it, and I tried to avoid it.” (002-P-140)
6. Something not to do/to reject/to avoid		
7. Something to postpone		
8. Lack of information in general	4. Lack of knowledge about PC among physicians and population	“Palliative care is a treatment that literally everyone identifies with death.” (002-C-050) “I don’t think anyone knows the difference between palliative care and early palliative care.” (002-C-058) “But to be honest the oncologist didn’t explain it correctly.” (002-C-070) “Well, as a doctor myself, I can say that physicians don’t know early palliative care and that they even consider it unnecessary or at least something they have to apologize for as if they are saying there is nothing more they can do.” (002-C-009) “A friend of mine went to a place to die and told me about this care.” (002-P-124)
9. Lack of information among the population		
10. Lack of information among the physicians		
11. Attribution of a new meaning of care of life/care of dignity	5. Acquired awareness of early PC meaning and benefits	“To me, palliative care was the equivalent of death (…). This was what I thought before coming here and before receiving it. Now I know this is not true.” (002-P-145) “I thought it was just the treatment they give you when you’re about to die, but then, when I came here, I realized I was wrong.” (002-C-063) “Then we learned that (…) early palliative care takes away the pain and allows to improve the quality of life and to be much more peaceful.” (002-C-059) “Early palliative care allowed me to resolve my great anguish, which was that of not being able to talk about my death. By coming here and talking about it with the doctor, I am more peaceful and even emotionally improved. I’m not saying I’m living without thinking about death, of course, but now I can accept to think about it.” (002-P-145) “And then I think that without this care my aunt would have certainly lived with a very bad quality of life, and she would have also lived much less time.” (002-C-046) “(…) it meant for us salvation and hope.” (002-C-058) “Now I am much more peaceful and my gratitude is huge.” (002-P-132) “If I hadn’t found this care, I wouldn’t be alive now, I had already decided to end it, I mean, to go to a clinic in Switzerland to ask for euthanasia. Now I think I would have done the most wrong thing of my life.” (002-P-140) “This care is what all cancer patients should do immediately, because both the patient and the family enormously benefit from it.” (002-C-106)
12. Impact on life in general		
13. Impact on quality of life		
14. Impact on physical and/or psychological pain		
15. Impact on the idea of death		
16. Feeling of gratitude		
17. Alternative to euthanasia		
18. Acquired awareness of differences with standard PC	6. Awareness of the differences between early PC and late PC	“Then we learnt that it is the opposite of palliative care; this a cure for life and its quality: care for life instead of care for death.” (002-C-059) “Well, in short, this care is very different from the idea we had before we come here.” (002-C-041) “We did not know palliative care before the oncologist proposed it to us. But once he explained to us that it was intended for pain, we immediately accepted it, discovering later that it was much more than pain therapy.“ (002-C-055)
19. Regret for not starting earlier	7. Regret for the delayed referral	“I didn’t know about early palliative care, as we later got to know, but unfortunately, due to the fear evoked by this name, too late. We should have come here earlier.” (002-C-056) “And now I’m very sorry for not coming here earlier.” (002-C-108)
20. Regret for wasting time pondering		
21. Belief that the name is misleading	8. Name as a barrier	“I would change the name, because my family and friends, when I say I attend the early palliative care unit, get worried. Therefore, I prefer to say that I attend the supportive care unit or that I meet the pain doctor. I think this makes them feel more relaxed.” (002-P-137) “I would propose to change the name, because ‘palliative care’ scares everyone.” (002-C-008)
22. Suggestion to call it supportive care/pain care		
23. Belief that it should be mandatory/offered to everyone	9. Need to include early PC in the clinical routine	“If the benefits of this care would be known, everyone would ask for it.” (002-C-017) “(…) which should be proposed to every patient as soon as possible.” (002-C-001) “Moreover, I think that they should be mandatory for all patients with cancer and symptoms, so there would be no fear related to the name.” (002-C-070) “I think they should be mandatory so that the patient comes early. as any other routine visit.” (002-P-146)
24. Belief that everyone would request it if they knew about it		

Legend: at the end of each quotation the ID of the participant is reported: the first three numbers indicate the unit (002 for the Oncology and Palliative Care Unit, Civil Hospital Carpi), the letter indicates patient (P) or caregiver (C), and the last three numbers indicate the recruitment progressive number.

## Data Availability

The data underlying this article will be shared upon reasonable request to the corresponding author.
